# Ion Conductivity of the Bacterial Translocation Channel SecYEG Engaged in Translocation*

**DOI:** 10.1074/jbc.M114.588491

**Published:** 2014-07-11

**Authors:** Denis G. Knyazev, Lukas Winter, Benedikt W. Bauer, Christine Siligan, Peter Pohl

**Affiliations:** From the Institute of Biophysics, Johannes Kepler University Linz, Gruberstrasse 40, A-4020 Linz, Austria

**Keywords:** Electrophysiology, Lipid Bilayer, Membrane Transport, Membrane Transporter Reconstitution, Protein Translocation, Proton Motive Force

## Abstract

While engaged in protein transport, the bacterial translocon SecYEG must maintain the membrane barrier to small ions. The preservation of the proton motif force was attributed to (i) cation exclusion, (ii) engulfment of the nascent chain by the hydrophobic pore ring, and (iii) a half-helix partly plugging the channel. In contrast, we show here that preservation of the proton motif force is due to a voltage-driven conformational change. Preprotein or signal peptide binding to the purified and reconstituted SecYEG results in large cation and anion conductivities only when the membrane potential is small. Physiological values of membrane potential close the activated channel. This voltage-dependent closure is not dependent on the presence of the plug domain and is not affected by mutation of 3 of the 6 constriction residues to glycines. Cellular ion homeostasis is not challenged by the small remaining leak conductance.

## Teaser

**Background:** How SecYEG excludes ions during protein translocation is not known.

**Results:** SecYEG gating is voltage-sensitive.

**Conclusion:** Voltage minimizes the ion leak that is induced by stalling a translocation intermediate.

**Significance:** Preservation of the proton motif force requires voltage-driven conformational changes.

## Introduction

After being synthesized in the cytosol, many proteins need to be either (i) inserted into the plasma membrane or the membrane of the endoplasmic reticulum or (ii) transported across them (1). A major pathway in bacteria is provided by the heterotrimeric protein translocation channel SecYEG (translocon or SecY complex) (2). Its structurally conserved family member, the eukaryotic Sec61 complex (3), is located in the membrane of the endoplasmic reticulum.

The complexes must prevent proton or calcium leakages because collapses of (i) the proton motif force across the inner bacterial membrane or (ii) the Ca^2+^ concentration gradient across the membrane of the endoplasmic reticulum are incompatible with life. The pore ring, a hydrophobic constriction zone in the middle of the channel, and the plug, a mobile reentrant loop in the periplasmic half of the funnel, seal the resting channel (4).

It is not known how the prokaryotic and eukaryotic translocons prevent H^+^ or Ca^2+^ leakages during protein transport. The plug is moved out of the pathway for secretory proteins so that ions may also permeate (5). The pore ring is envisioned to form a gasket-like seal around the translocating protein (6). However, the very flexible belt of six hydrophobic amino acids may adopt any diameter between 4 and 24 Å (7). It is thus a rather unorthodox ion selectivity filter. For example, cation and proton exclusion by water channels (8, 9) requires a strong electrostatic field in an extremely narrow restriction zone that is created by a charged amino acid and by dipoles of reentrant loops (10).

Here we present mechanistic insight into how SecYEG manages to maintain the barrier to ions and protons during protein translocation. We show that binding of a translocation intermediate opens a large SecYEG pore only at small transmembrane potentials. Physiological membrane potentials close the channel. With a stalled polypeptide chain, the unitary leak conductance of the closed channel is about 2 orders of magnitude smaller than the conductance of the open SecYEG.

## MATERIALS AND METHODS

### 

#### 

##### Signal Peptide

The signal peptide from the precursor form of outer membrane protein A (leader peptide (LP))^3^ contained a cysteine instead of an alanine in the last position (sequence: MKKTAIAIAVALAGFATVAQC). This peptide was synthesized and purified to 98% purity by Peptide 2.0 Inc. (Chantilly, VA).

##### Translocation Intermediate

The preprotein construct (pOD) consisted of the precursor form of outer membrane protein A, proOmpA, which was truncated at position 69 (11, 12) and connected at its C terminus to the cytosolic enzyme dihydrofolate reductase. pOD was expressed in MM52 temperature-sensitive cells. 100 ml of 2× YT culture of transformed MM52 cells, supplemented with 100 μg/ml ampicillin, were grown at 30 °C to an optical density of 1 (measured at a wavelength of 600 nm). The culture was then added to 900 ml of prewarmed 2× YT medium, supplemented with 100 μg/ml ampicillin, and grown at 37 °C for another 30 min. Overexpression was induced by the addition of 2 g/liter arabinose, and cells were harvested after 2 h of growth at 37 °C. Cells were lysed in buffer containing 50 mm Tris, pH 7.5, 300 mm KCl, 10% glycerol, 1 mm tris(2-carboxyethyl)phosphine, and cOmplete protease inhibitor mixture (EDTA-free, Roche Applied Science) using an EmulsiFlex C-5 (Avestin) at 20,000 p.s.i. The soluble fraction of the lysate was isolated by ultracentrifugation for 60 min at 40,000 rpm, 4 °C (Beckmann Ti90 rotor). The supernatant was incubated with nickel-nitrilotriacetic acid resin (Qiagen) for 60 min at 4 °C. Bound protein was washed in the presence of 20 mm imidazole, eluted with buffer containing 50 mm Tris, pH 7.5, 100 mm KCl, 10% glycerol, and 300 mm imidazole, and stored at −80 °C.

##### SecA Purification

SecA was overexpressed in *Escherichia coli* C43(DE3) from the pET30 SecA expression vector and induced by 1 mm isopropyl-1-thio-β-d-galactopyranoside at 37 °C (13). After 3 h, the cells were pelleted and lysed by a homogenizer in 0.5 m NaCl, 20 mm Tris (pH 7.5) using two cycles of 20,000 p.s.i. After centrifugation for 90 min at 40,000 rpm and 4 °C, the supernatant was incubated with equilibrated Ni^2+^-chelating beads for 1 h at 4 °C. The beads were loaded on a column and washed in the presence of 20 mm imidazole. SecA was eluted with 200 mm imidazole and then subjected to size exclusion chromatography using 100 mm NaCl, 20 mm Tris (pH 7.5), and 2 mm β-mercaptoethanol.

##### SecYEG Purification

Expression vectors for SecY complexes were based on the pBAD-SecYEG cysteine-less mutant containing an A204C substitution for labeling. In the plug-less SecY mutant (SecΔP), amino acids 60–74 have been replaced by amino acids GSGS, and the plug-less SecY triple ring mutant (SecΔP3G) additionally contained substitutions I86G, I191G, and I278G. All SecY complexes were purified from *E. coli* C43(DE3) cells after 4 h of induction with 0.2% arabinose at 37 °C as described (4). This included protein extraction by dodecyl-β-d-maltopyranoside (Anatrace), overnight incubation with Ni^2+^-chelating beads, and size exclusion chromatography.

##### Protein Reconstitution into Lipid Vesicles

The freshly purified SecY complexes were reconstituted into proteoliposomes using Bio-Beads SM2 (Bio-Rad) for detergent removal (4). In brief, the reconstitution mixture was prepared at room temperature by sequentially adding 20 mg/ml *E. coli* polar lipid extract (Avanti Polar Lipids, Alabaster, AL) in 50 mm K-HEPES, pH 7.5, 6% deoxy-Big-CHAP (Affymetrix Anatrace, Cleveland, OH) and purified protein in detergent (protein-to-lipid ratio of 1:36 to 1:100). Subsequent to detergent removal by Bio-Beads, the proteoliposomes were harvested by ultracentrifugation (80 min at 100,000 × *g*) and resuspended in an assay buffer at a lipid concentration of 5–10 mg/ml. The assay buffer contained 50 mm HEPES, pH 7.0, 10% glycerol and protease inhibitor.

##### Reconstitution of the SecY Complex into Planar Bilayers

“Solvent-free” planar bilayers were folded by raising the level of two adjacent aqueous solutions over the dividing aperture in a Teflon septum with *E. coli* polar lipid extract (Avanti Polar Lipids, Alabaster, AL) monolayers on top (14). Fusion of proteoliposomes containing the corresponding SecY complex at protein-to-lipid mass ratio between 1:36 and 1:100 with the free-standing planar lipid membranes was facilitated by a 450 mm:150 mm KCl gradient across the planar bilayer (15, 16). If a SecYEG channel was open in the vesicular membrane, the osmolyte entered the respective vesicle. Water from the hypotonic (*trans*) compartment followed when this vesicle adhered to the planar bilayer. Subsequent vesicle swelling resulted in fusion with the planar bilayer, thus allowing detection of current flow through open SecYEG channels.

To allow pOD to open the translocon, the *cis* compartment contained 650 nm *E. coli* SecA, 1 mm MgCl_2_, and 0.8 mm ATP. The *cis* compartment also harbored the proteoliposomes. Both compartments were buffered by 50 mm K-HEPES at pH 7.5. To prevent aggregation of the signal peptide, 90 mm urea was also added to the *cis* compartment in the respective experiments.

##### Single Ion Channel Measurements

Single channel measurements were performed as described previously (17, 18). Ag/AgCl reference electrodes in the *cis* and *trans* compartments were connected to command signal of the patch clamp amplifier to the command signal of the patch clamp amplifier (model EPC9, HEKA Electronics) and the ground, respectively. The recording filter for the transmembrane current was a 4-pole Bessel with −3 dB corner frequency of 0.1 kHz. The raw data were analyzed using the TAC software package (Bruxton Corp., Seattle, WA). Gaussian filters of 12 or 112 Hz were applied to reduce noise.

## RESULTS

**FIGURE 1.**
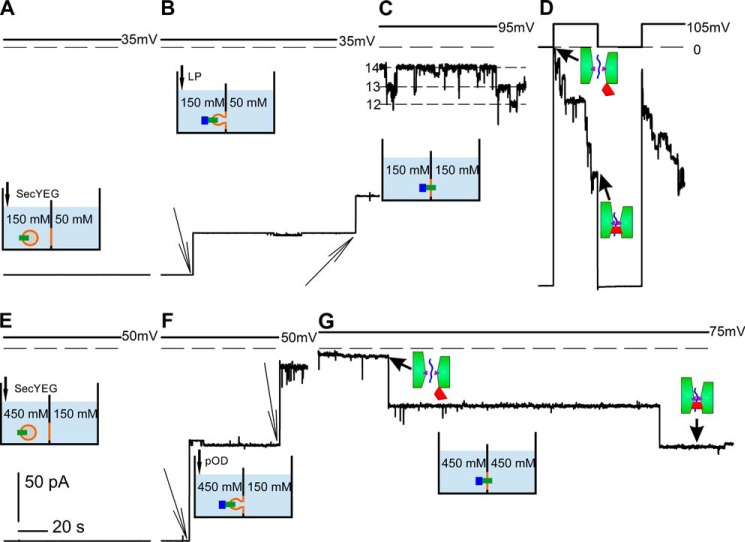
**Openings of the SecYEG channel in response to bindings of the LP of proOmpA or a longer preprotein construct (pOD).** *A–D,* response to LP. *A*, bilayer background conductivity before LP addition. The transmembrane KCl gradient amounted to 150:50 mm KCl. Both compartments contained 10 mm K-HEPES (pH 7.5). The membrane potential was clamped at −35 mV. *B*, peptide addition (100 nm) opened SecYEG channels in the vesicular membranes. Subsequent proteoliposome fusion to the bilayer lipid membranes (shown with *thin arrows*) resulted in large current steps. *C*, the addition of KCl to the hypotonic compartment stopped vesicle fusion. The membrane potential was clamped at +95 mV. *D*, we observed stepwise channel closings of SecYEG at +105 mV. The single channel conductance *g* was equal to 220 ± 20 pS. *E–G*, response to pOD. *E*, bilayer background conductivity before peptide addition. The aqueous solutions contained SecYEG proteoliposomes plus 450 mm KCl on one side and 150 mm KCl on the other. Both compartments were buffered with 50 mm K-HEPES (pH 7.5). The *cis* compartment contained 650 nm SecA, 1 mm MgCl_2_, and 0.8 mm ATP. The membrane potential was clamped at −35 mV. *F*, the addition of 100 nm pOD led to proteoliposome fusion with the bilayer lipid membranes. In turn, the transmembrane current increased stepwise. The membrane potential was clamped at +50 mV. *G*, the addition of KCl to the hypotonic compartment stopped vesicle fusion. The membrane potential was clamped at +75 mV. As expected from the 3-fold higher salt concentration (compare *C* and *D*), *g* was equal to ∼700 pS.

We formed planar bilayer lipid membranes and added SecYEG-containing proteoliposomes to the *cis* compartments. Subsequently, we raised the osmolarity in that compartment. If the SecYEG were open, the vesicles would fuse with the planar bilayer and, in turn, the thus inserted SecYEG would give rise to transmembrane current fluctuations. Because the resting SecYEG is closed (4), we did not detect channels, *i.e.* fusion did not occur and the membrane retained its low conductance state (Fig. 1*A*). Likewise, the addition of the dissolved 21-amino acid-long LP of proOmpA to the pure lipid bilayer did not induce channel activity, neither under isosmotic nor under hyperosmotic conditions.

However, the simultaneous presence of proteoliposomes and LP in the hyperosmotic compartment resulted in channel activity (Fig. 1*B*). That is, LP opened SecYEG in the vesicles, thereby enabling fusion of the vesicles to the planar bilayer. This resulted in current flow through open SecYEG channels. Because the vesicles contained several SecYEG copies, every fusion event was indicated by a large current step (Fig. 1*B*).

Subsequent closure of the SecYEG channels resulted in a stepwise decrease of the transmembrane current (Fig. 1, *C* and *D*). The LP from proOmpA opened the translocon for longer periods of time than was reported for the LamB LP bound to microsomal membranes (19).

We performed at least 20 more experiments and repeatedly observed the same picture; membrane current had its maximal value immediately after vesicle fusion. This conductance level was relatively stable at moderate membrane potentials (Fig. 1*C*) and declined at higher membrane potentials (Fig. 1*D*). We concluded that channel closure was driven by membrane potential.

When substituting the short LP for the longer pOD, the open probability of SecYEG significantly increased in the presence of SecA, as indicated by a 10-fold larger membrane current. To observe single channels (Fig. 1, *F* and *G*), we decreased the SecYEG concentration accordingly. Most interestingly, SecYEG now appeared to be more sensitive to external voltage. That is, we observed channel closure at smaller membrane potentials (Fig. 1*G*).

**FIGURE 2.**
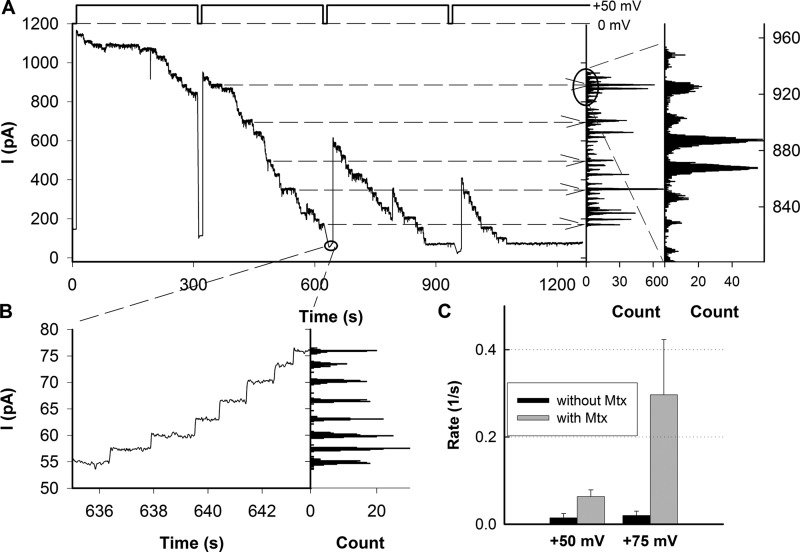
**SecYEG closure is driven by application of a transmembrane voltage ψ*_a_*.** *A*, ψ*_a_* is shown on top of the experimental records. The *upper trace* shows consecutive channel closings for ψ*_a_* = +50 mV. Histogram analysis and the zoom-in (*right panel*) show a single channel current of 27.5 ± 2.4 pA. The 450–150 mm KCl transmembrane concentration gradient and the anion selectivity of SecYEG gave rise to a reversal potential, ψ_rev_. ψ_rev_ ∼7 mV results in a total membrane potential ψ = ψ_a_ − ψ_rev_ = ∼57 mV and in *g* of ∼480 ± 45 pS, respectively. Doubling of *g* (compare Fig. 1, *C* and *D*) corresponds well to the 2-fold increase in average salt concentration. *B*, zoom-in into the record of the upper panel. The current throughout the reopening SecYEG channels at ψ*_a_* = 0 mV was driven by ψ_rev_. The hypertonic compartment contained 100 nm pOD, 650 nm SecA, 1 mm MgCl_2_, 0.8 mm ATP, and 9 μm Mtx. Both compartments were buffered with 50 mm K-HEPES (pH 7.5). *C*, the frequency of channel closure increased with voltage and the addition of Mtx. It remained unaltered by plug and ring mutations so that the combined data for SecYEG, ΔP, and ΔP3G are shown (compare Fig. 3). *Error bars* indicate mean + S.D.

Complete translocation of pOD may explain the change in voltage sensitivity as it would be accompanied by dissociation of the leader sequence from its SecY binding site. To test this assumption, we repeated the experiment in the presence of methotrexate (Mtx). Its binding to dihydrofolate reductase prevents unfolding of the enzyme. As a result, pOD is unable to permeate the channel, and a true translocation intermediate is formed (11). Binding of the pOD-Mtx complex to SecYEG opened the channel. Mtx significantly accelerated closure, whereby the rate was voltage-dependent (Fig. 2*C*). A small leak current *I*_L_ remained (∼65 pA in Fig. 2*A*) that could be attributed to the closed channels with a stalled translocation intermediate (as shown below). Moreover, the presence of Mtx rendered channel closure reversible (Fig. 2*B*). That is, when the bilayer lipid membrane was clamped to 0 mV, most of the channels reopened one after the other. As indicated by *I*_L_, the reconstituted SecYEG channels were still occupied by translocation intermediates. Consequently, these still bound intermediates must have triggered the reopenings.

On the contrary, without Mtx, pOD translocation into the *trans* compartment has most probably taken place because most of the channels lost the polypeptide during the transition from the open to the closed state, and therefore only a small fraction of them reopened under these conditions. The only alternative explanation that pOD has dissociated back into the *cis* compartment is not very plausible because the probability of this event should not depend on the presence of Mtx. That is, Mtx does not hamper the dissociation of pOD from the translocon, but only its translocation.

Likewise, the response to voltage did not depend on Mtx. SecYEG closed at the same small potentials as if no Mtx was bound (Fig. 2*A*). We conclude that voltage sensitivity is not due to a voltage-driven release of the peptide from SecYEG.

**FIGURE 3.**
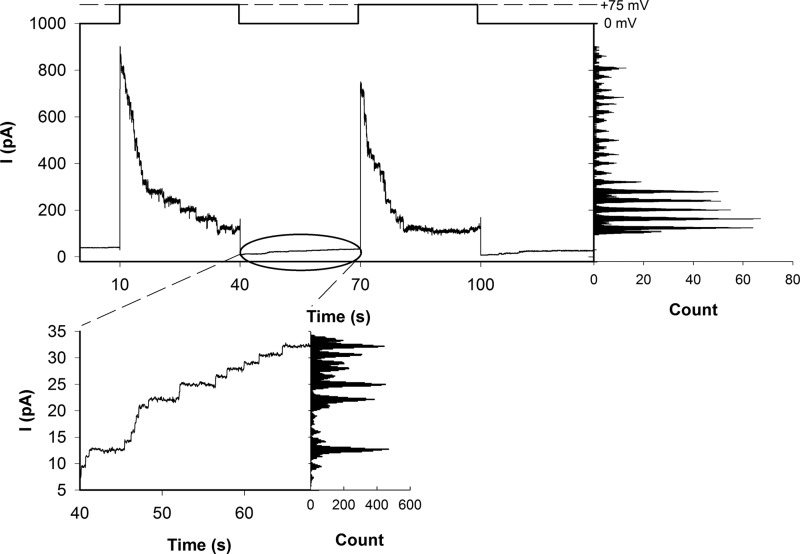
**Voltage-driven closure of ΔP3G.** The experimental conditions were as in in Fig. 2. Assuming ψ_rev_ = −3.5 mV (compare Fig. 4) results in a total membrane potential ψ = ψ_a_ +ψ_rev_ and *g* of 53.4 mV and ∼476 pS, respectively. The amplitude of channel reopenings at ψ*_a_* = 0 mV was equal to ∼1.5 ± 0.6 pA.

In an attempt to identify the sensor, we (i) removed the plug (ΔP) and (ii) substituted three isoleucines of the hydrophobic ring by hydrophilic glycines (ΔP3G). If the plug was the voltage sensor, we expected ΔP to remain open at large membrane potentials. If any other charged residue (i) in close vicinity to the pore ring or (ii) on the spontaneously forming new plug (20) was the sensor in ΔP3G, it must have been exposed to a decreased voltage drop per unit channel length. That is, instead of dropping in the very narrow region of the hydrophobic ring as in ΔP, the electric field drops over a longer distance in ΔP3G because its constriction site is dilated and contains ions as indicated by spontaneous channel activity of pore ring mutants (4). However, the voltage sensor did not respond to the decreased strength of the electric field in the constriction zone. ΔP3G and ΔP closed with identical voltage sensitivities. Similar to the wild type channel (Fig. 2), both ΔP and ΔP3G reopened in the presence of Mtx when no external potential was applied (Fig. 3). We concluded that neither plug nor pore ring was primarily important for maintaining the membrane barrier to ions during translocation. Instead, the voltage-driven conformational change of SecYEG was crucial for blocking ion permeation.

**FIGURE 4.**
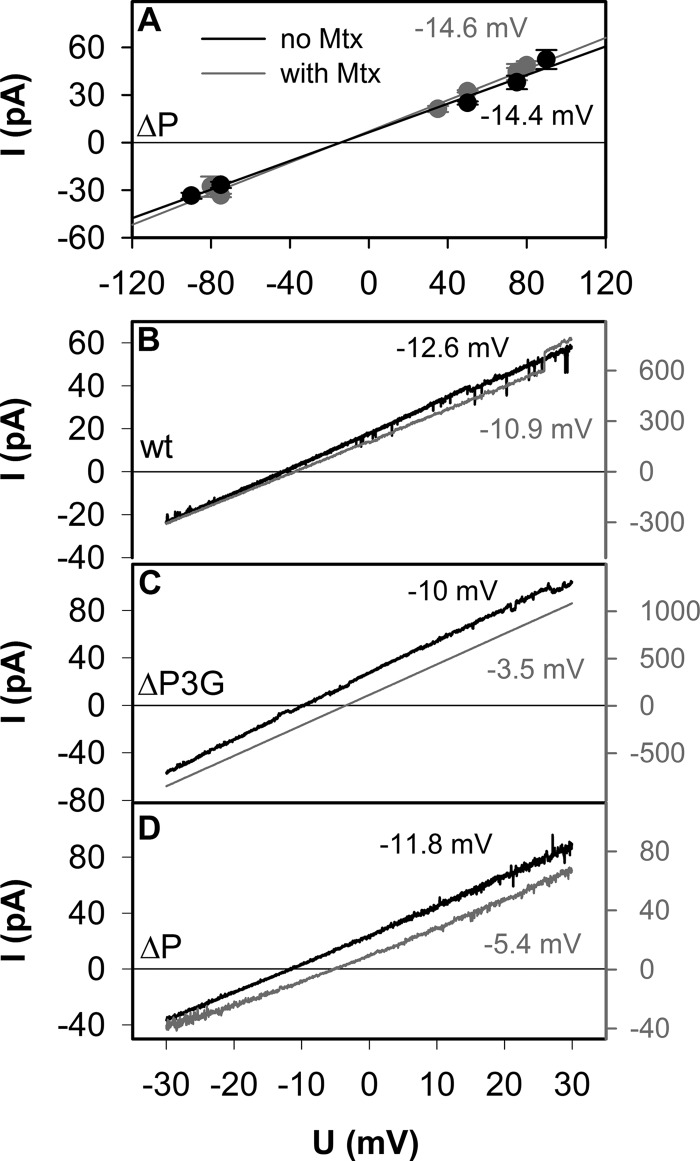
**Ion selectivity of SecYEG (*B*), ΔP3G (*C*), and ΔP (*A* and *D*), opened by a permeable pOD construct (*black*) and a stalled (*gray*) translocation intermediate (pOD-Mtx).** *A*, the voltage dependence of the single channel current shows that Mtx does not alter channel selectivity. Single channel conductivity for pOD construct in the absence and presence of Mtx is 451 ± 50 and 491 ± 59 pS, respectively. *B–D*, ψ*_a_* changed continuously at a rate of 6 mV/s between −30 and +30 mV. Corresponding reversal potentials are given in mV. For experimental conditions, see Fig. 2.

In the absence of an externally applied potential, the current through the channels was driven by the reversal potential, ψ_rev_. Generation of ψ_rev_ in a salt gradient (450:150 mm KCl) indicated that SecYEG must possess ion selectivity. It can be quantified in terms of the anion to cation permeability ratio, *P*_Cl−_/*P*_K+_. The voltage dependence of the single channel current of ΔP indicated a ψ_rev_ of −14.6 mV (Fig. 4*A*, *black line*). According to the textbook equation for the resting membrane potential,

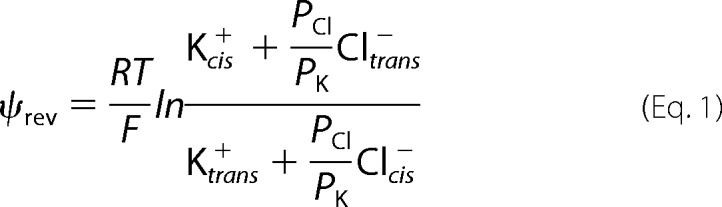
 it corresponds to *P*_Cl−_/*P*_K+_ ≈ 3.7, where the subscripts *cis* and *trans* indicate the two sides of the membrane.

When measuring the voltage dependence of the current across the whole membrane, we found ψ_rev_ ≈ −12.6, −10.0, or −11.8 mV (Fig. 4, *B*, *C*, and *D*, respectively, *black curves*) for membranes that contained the wild type channel, ΔP3G or ΔP, respectively. This indicated that the reconstituted bilayers possessed a smaller selectivity of potassium over chloride than the translocon proper. This observation suggested the presence of a leak current *I*_L_ in addition to the current through the open SecYEG. Closed SecYEG channels with stalled pOD represented the most likely pathway for *I*_L_. To test this hypothesis, we augmented the relative amount of closed channels with stalled translocation intermediates by allowing Mtx binding to pOD. The experiment confirmed our prediction; Mtx decreased ψ_rev_ for membranes that were reconstituted with the wild type channel, with ΔP3G, or with ΔP (Fig. 4, *B*, *C*, and *D*, respectively, *gray curves*). In contrast, control single channel experiments revealed the same ψ_rev_ in both the presence and the absence of Mtx (Fig. 4*A*). Thus, *I*_L_ measurements corroborate the conclusion about pOD translocation in the absence of Mtx that was based on irreversible channel closure at elevated voltages.

**FIGURE 5.**
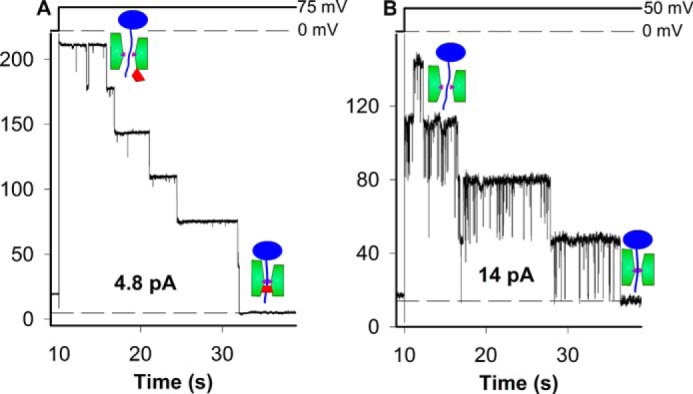
**Closed SecYEG (*A*) or ΔP (*B*) channels that contain stalled translocation intermediates allow for small nonselective leak currents.** *A* and *B*, leakage per channel amounts to 0.8 (*A*) or 3.5 pA (*B*). For buffer solutions, see Fig. 2.

We next proceeded to measure *I*_L_. We obtained a current of 4.8 pA after closure of six wild type channels with stalled pOD-Mtx, *i.e. I*_L_ amounted to 0.8 pA per SecYEG complex (Fig. 5*A*). For ΔP, *I*_L_ per closed channel was equal to 3.5 pA, *i.e.* it was roughly four times larger than for the wild type channel (Fig. 5*B*). At about 4.3 pA, *I*_L_ per channel was maximal for ΔP3G.

## DISCUSSION

We have shown that the voltage-driven closure of the SecYEG pore preserves the membrane barrier to ions, and thus, the proton motif force. That is, at physiologically relevant values of ionic strength and the typical bacterial plasma membrane potential ψ_G_ = −130 mV (21), the total leakage current per translocon that contains a stalled translocation intermediate is negligibly small (∼0.8 pA). The corresponding conductivity of 6 pS is 2 orders of magnitude smaller than the current through the ΔP mutant (4) or the open SecYEG (16).

The voltage dependence explains the viability of ΔP mutants (22) and prlA4 mutants. Both are known to provide a pathway to ions at small membrane potentials (4), but appear to be nearly closed at physiologically relevant values of the membrane potential (Fig. 3). Voltage-driven conformational changes also solve the apparent contradiction between the observation that ribosome binding opens SecYEG channels (16) at small membrane potentials and the lack of a significant ion or proton leak across the bacterial plasma membrane under physiological conditions.

Our conclusion that voltage-driven channel closure is responsible for the preservation of proton and ion gradients across the bacterial plasma membrane is in line with the reported lack of Cl^−^ or xylitol leakage through wild type SecYEG that is occupied with a nascent chain, as measured with spheroplasts (6). The observation that ΔP mutants showed Cl^−^ leakage only when treated with valinomycin (6) suggested anion selectivity (23, 24). Because we determined *P*_Cl−_/*P*_K+_ ≪ 100, we can now rule out that anion selectivity is crucial for maintaining the proton motif force. For example, a Cl^−^ concentration increase of 3 mm would translate into a cytoplasmic pH drop of 1 unit assuming (i) P_CL-_/P_H+_ ≈ 3.4 and (ii) characteristic values of buffer capacity and protein content per cell, ∼50 μmol/mg of protein (25), and ∼20 mg (26), respectively.

The remaining question is how SecYEG closes in response to membrane potential, *i.e.* what exactly is the voltage-sensitive element? We probably can rule out the plug. It is true that plug removal is required for the observation of large ion conductivity (4). However, plug removal does not hamper the voltage-driven channel closure (Fig. 3). There is also no possibility that the replacement plug formed upon plug deletion (20) takes over the role. It carries a positively charged residue (Arg-57) instead of two oppositely charged amino acids (Glu-62, Arg-74) in the original plug. If the replacement plug was the voltage sensor, the closure rates for the wild type and plug-less mutants should not have been similar (Fig. 2*C*).

**FIGURE 6.**
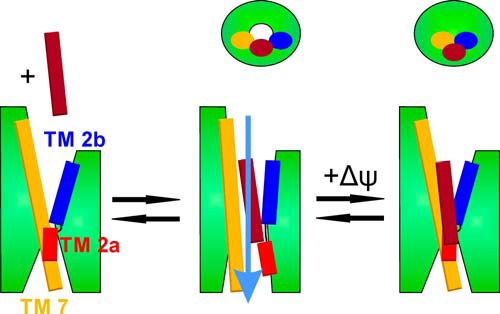
**Putative scheme of voltage-driven channel closure.** The resting channel is closed (*left panel*). LP binding opens the translocon (*middle panel*). It becomes permeable to the translocating peptide chain as well as to ions and water (represented by *blue arrow*) in the absence of ψ*_a_*. Physiological values of ψ*_a_* close the channel (*right panel*). The stalled translocation intermediate is likely to be pushed to the lipid side of the lateral gate. The *upper panel* shows the channel from the cytoplasm. *TM*, transmembrane helix.

Thus, unless the movements of the original and the replacement plugs are not accompanied by dramatic p*K_a_* shifts of the amino acid side chains, some other structural element must act as the voltage sensor. This sensor must respond to both positive and negative voltages with the same response: channel closure. The rotation of a helix involved in pore ring formation would be an option. Voltage-driven closure of the lateral gate would be another possibility (compare Fig. 6). LP or the N terminus of pOD would reside on the lipid side of the lateral gate, reminiscent of what has been observed in crystal structures of the SecYEG-ribosome or Sec61-riboosme complexes with a stalled translocation intermediate (27, 28). Further experiments are required to test these hypotheses.

We conclude that preservation of the bacterial proton motif force is due to voltage-driven closure of the translocation channel. Neither plug deletion nor pore ring mutations are able to significantly disturb the gating. Experiments devoted to the identification of the voltage-sensitive element are under way.

## Footnotes

3 The abbreviations used are:

LP: leader peptide
proOmpA: outer membrane protein A
Mtx: methotrexate
pS: picosiemens
pOD: proOMPA-DHFR construct.
